# AmpliRAD: A New Method Combining Amplicon and RAD Sequencing

**DOI:** 10.1002/ece3.72990

**Published:** 2026-02-11

**Authors:** Tasha Q. Thompson, Michael R. Miller, Matthew R. Sloat

**Affiliations:** ^1^ Wild Salmon Center Portland Oregon USA; ^2^ Department of Animal Science University of California Davis California USA

## Abstract

Reduced‐representation sequencing methods, such as Restriction‐site Associated DNA sequencing (RAD‐seq), use restriction enzymes to achieve a cost‐effective approach for generating genome‐wide SNP data. However, a major limitation of these methods is their inability to directly assay specific loci of interest unless located near restriction sites. Here, we present ampliRAD, a novel method combining targeted (i.e., amplicon) and reduced‐representation sequencing. AmpliRAD uses an initial multiplex PCR step to amplify target loci and append restriction enzyme recognition sites onto them. The PCR product is then combined with genomic DNA and used as input for a traditional RAD library preparation protocol, enabling the incorporation of virtually any target loci into a standard RAD dataset. We also introduce updates to an existing RAD protocol, including enzymatic shearing, that enhance its accessibility and efficiency. To demonstrate ampliRAD's utility, we investigate genetic associations with adult migration timing in Dean River Chinook salmon, revealing a clear link between the *GREB1L* locus and migration timing that extends previous findings from southern populations to this northern river. AmpliRAD provides a powerful new tool for genomic analyses, offering the combined benefits of both reduced representation and targeted sequencing approaches.

## Graphical abstract

We present AmpliRAD, a novel sequencing method that integrates a multiplex PCR step into traditional RAD‐seq protocols to enable the simultaneous generation of genome‐wide SNP data and targeted genotypes for specific loci. By appending restriction sites to target amplicons, the method overcomes the inability of standard RAD‐seq approaches to assay loci not located near natural restriction sites. We demonstrate the utility of this method in Dean River Chinook salmon, revealing a significant association between the GREB1L locus and adult migration timing while concurrently resolving fine‐scale population structure.

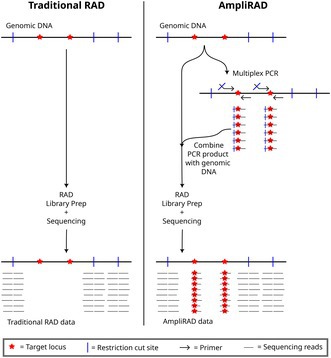

## 1 Introduction

Rapid advances in genetic sequencing technologies, particularly the development of massively parallel sequencing (MPS), also known as next‐generation sequencing (NGS), have revolutionized nearly all facets of biological research (Satam et al. 2023; Shendure and Ji 2008; Shokralla et al. 2012). By enabling the simultaneous sequencing of millions or billions of DNA fragments, MPS has exponentially increased the scale and dramatically reduced the cost of genetic analyses. Generating terabytes of data is now achievable even for projects with relatively modest budgets, democratizing genomic analyses and facilitating research in non‐model organisms (Ekblom and Galindo 2011; Hohenlohe et al. 2021).

While current MPS methods offer the potential for comprehensive genomic data generation (i.e., whole‐genome sequencing) across numerous samples (Fuentes‐Pardo and Ruzzante 2017; Lou et al. 2021; Satam et al. 2023), the resulting deluge of data presents significant challenges. These challenges include the substantial computational resources required for analyses, the time necessary for processing, and the escalating costs of data storage. In many cases, however, comprehensive genomic data are not necessary to address biological questions of interest. Reduced‐representation and targeted sequencing approaches often provide sufficient information while mitigating many of the computational, financial, and logistical burdens associated with whole‐genome sequencing (Campbell et al. 2015; Fuentes‐Pardo and Ruzzante 2017).

Reduced‐representation sequencing generates data from a subset of genomic DNA. A common approach is Restriction‐site Associated DNA sequencing (RAD‐seq) and its derivatives (Andrews et al. 2016; Baird et al. 2008; Miller et al. 2007). RAD‐seq involves digestion of genomic DNA with a restriction enzyme, followed by library preparation that preferentially enriches for DNA fragments adjacent to the restriction sites (RAD tags). Because restriction sites are typically distributed relatively randomly across a genome, RAD‐seq offers a relatively unbiased, reduced representation of genome‐wide data suitable for diverse analyses. Depending on the restriction enzyme used, these methods can generate tens of thousands to millions of RAD tags per sample, enabling studies of fine‐scale population structure, genetic associations with adaptive traits, and other applications (Andrews et al. 2016; Narum et al. 2013). However, the limitation of analyses to loci located on RAD tags can constrain the utility of RAD‐seq. For example, RAD‐seq data cannot be used to directly assay trait‐associated markers that are not located proximal to a restriction site. In such cases, targeted sequencing methods are required to obtain genotypes at these specific loci.

Targeted sequencing enriches for pre‐defined loci, typically using either amplicon sequencing with locus‐specific primers or sequence capture with hybridization probes (Meek and Larson 2019). Of the two, amplicon sequencing is generally simpler and more common, particularly in non‐model organisms. Targeted sequencing is ideal for applications investigating specific loci, such as determining allele frequencies at adaptive markers. A targeted panel of putatively neutral loci can also efficiently address questions of parentage and broad‐scale population structure at very low sequencing cost (Campbell et al. 2015). However, developing informative primer or probe sets for neutral loci requires substantial initial investment of time and resources. Furthermore, the number of loci obtainable through targeted methods (typically up to a few hundred for amplicon sequencing) is far fewer than with RAD‐seq. This restricts the utility of targeted approaches for some analyses such as genome‐wide association studies and can limit the resolution of fine‐scale population structure. Ascertainment bias during panel development can also be a concern and may limit a panel's applicability across diverse populations (Lachance and Tishkoff 2013). Thus, while targeted approaches are well‐suited for routine, high‐throughput genotyping (e.g., in breeding or hatchery programs), reduced‐representation methods like RAD‐seq offer advantages in other contexts. Consequently, there are many applications where the advantages of both approaches may be desired.

**FIGURE 1:**
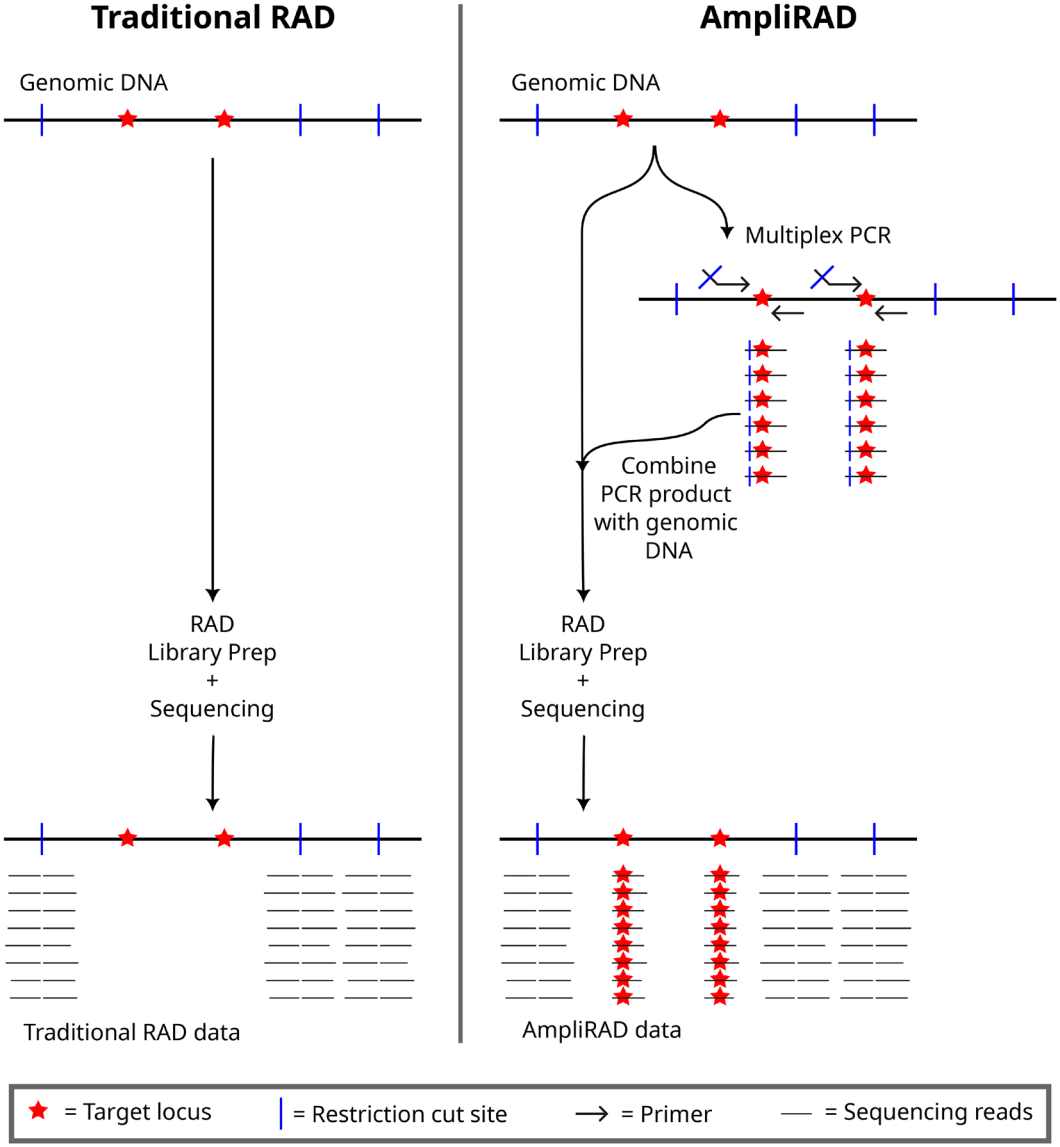
AmpliRAD versus traditional RAD data generation.

Here, we present ampliRAD, a novel method combining targeted and reduced‐representation sequencing (Figure 1). In ampliRAD, an initial multiplex PCR step amplifies target loci while also appending a restriction enzyme recognition site to each amplicon. This is accomplished by including a “tail” of DNA encoding a restriction site on the 5′ end of each forward primer. The PCR product is then mixed with genomic DNA from the same sample, and the mixture is used as input into a traditional RAD‐seq protocol, enriching for DNA adjacent to restriction sites. The resulting ampliRAD data includes sequences adjacent to naturally occurring restriction sites (as in traditional RAD‐seq) as well as the target sequences synthetically linked to restriction sites by the initial PCR step. To demonstrate the utility of ampliRAD, we apply the method to a case study in Dean River Chinook salmon, evaluating the association of adaptive markers discovered in other populations with phenotypic variation in the Dean River, then examining the findings within the context of population structure. Thus, ampliRAD offers all the benefits of RAD‐seq while overcoming a key limitation—the inability to directly assay specific loci of interest.

## 2 Methods

### 2.1 Sample Collection and DNA Extraction

Samples were collected from both adult and juvenile Chinook salmon from the Dean River, British Columbia. The adult samples were caught in the lower river recreational fishery by anglers from early June to mid‐August in 2022 and 2023. Juveniles were sampled by electrofishing in August of 2022. A small piece of caudal fin tissue was removed from each individual prior to live release back into the river. The tissue was dried on Whatman filter paper and stored in a coin envelope with information designating the date and location of sampling.

To extract DNA, a small piece (~1 mm square) of each sample was placed into a well of a 96‐well PCR plate. The tissue was digested with Proteinase K. DNA was extracted from the digested material according to an SPRI bead‐based protocol (Ali et al. 2016) and eluted into low‐TE buffer (10 mM Tris–HCl [pH 8.0] + 0.1 mM EDTA).

### 2.2 Study Design

DNA from 96 samples (55 adult Chinook salmon and 41 juveniles) was arrayed in a PCR plate for ampliRAD sequencing (Supporting Information). To facilitate comparison, an additional 13 juvenile samples were sequenced as part of another full plate using a traditional RAD protocol without the targeted (i.e., amplicon) component (Ali et al. 2016). The shared steps of the ampliRAD and traditional RAD protocols were performed simultaneously on both sets of samples. The two datasets will be referred to below as the ampliRAD and the traditional RAD sample/datasets.

### 2.3 AmpliRAD Protocol

Note: A document containing a detailed protocol with additional notes is provided in the Supporting Information.

#### 2.3.1 Target Primer Design

The first step in ampliRAD is the design of primers to target specific loci of interest. Here, we designed primers to amplify 39 target loci within the Chinook salmon genome (Supporting Information). The loci are situated on chromosome 28 within a region strongly associated with adult migration timing, a critical adaptive trait (Waples et al. 2022). Each target locus encompasses a single nucleotide polymorphism (SNP) of interest. The SNPs were identified in an analysis of Chinook salmon from across the species' range that will be presented elsewhere. Nineteen of the SNPs had also been identified in previously published analyses (Koch and Narum 2020; Prince et al. 2017; N. F. Thompson et al. 2020; T. Q. Thompson et al. 2019) (Supporting Information). Two of the SNPs are adjacent to naturally occurring *SbfI* restriction sites and were included to facilitate comparison with samples prepared using the traditional RAD protocol (Prince et al. 2017). The other 37 SNPs are not located near natural restriction sites.

Candidate primer pairs were identified with NCBI's Primer BLAST software (Ye et al. 2012). Approximately 500 bp of sequence centered around each target SNP was used as input for Primer‐BLAST. Minimum and maximum PCR product sizes were set to 250 and 320 bp, respectively (in order to match our typical target range for RAD library size), but the range was sometimes expanded to improve primer search results (the range of PCR product sizes for our final primer panel was 180–325 bp). Minimum and maximum primer melting temperatures (*T*_m_) were set to 57°C and 61°C, respectively, with an optimum of 59°C. Primer pair specificity was checked against the Chinook salmon reference genome (Otsh_v2.0) from the Refseq representative genomes database (Christensen et al. 2018). Default settings were used for all other Primer‐BLAST parameters.

Final primer pairs for each target locus were selected based on three criteria: (1) strict avoidance of known polymorphic sites (based on available whole‐genome data) within the primer sequences; (2) positioning of the target SNP within the first 150 bp of the amplicon to avoid potential decreases in coverage due to the downstream fragmentation step (this criterion was considered flexible); and (3) minimization of the potential for off‐target amplification. This last point was prioritized by selecting primers with multiple mismatches to off‐target regions, ideally near their 3′ end. However, certain sections of the target region on chromosome 28 pose challenges to highly specific primer design (e.g., due to limited differences from off‐target regions). Therefore, for targets where primer specificity was expected to be low or moderate, we focused on selecting primer pairs whose most likely off‐target PCR products (based on Primer‐BLAST results) would clearly align better to their own region of the genome than to our target region (e.g., because the ~300 bp of sequencing data from off‐target PCR products would contain multiple mismatches to the target region). In other words, where *primer* specificity was low, we focused on ensuring *alignment* specificity remained high in order to prevent off‐target PCR products from confounding downstream analysis of target‐loci data.

After primer sequences were designed, a sequence containing a four base‐pair leader followed by the *SbfI/PstI* restriction enzyme recognition sequence was added onto the 5′ end of each forward primer (5′‐ACGTCCTGCAGG–forward_primer_sequence‐3′; Figure 1; Supporting Information). The finalized primers were ordered from Integrated DNA Technologies (IDT) at 100 μM concentration in IDTE Buffer pH 8.0 (10 mM Tris–HCl/0.1 mM EDTA). Primers were not prepooled.

#### 2.3.2 Multiplex PCR to Amplify Target Loci

To amplify target loci and append restriction sites in the ampliRAD sample set, a working primer stock was prepared by combining all target primers and diluting them in low‐TE buffer (10 mM Tris–HCl [pH 8.0], 0.1 mM EDTA) to a final concentration of 0.5 μM for each primer. For each PCR reaction, the following components were combined: 5 μL Platinum Multiplex PCR Master Mix (Applied Biosystems 4464268), 2 μL of the combined primer working stock, and 3 μL of genomic DNA eluted in low‐TE buffer (variable concentrations). PCR was performed with a heated lid using the following cycling parameters:

**Table:** 

PCR program		
95°C	5 min	
95°C	30 s	(35 cycles)
57°C	90 s	
72°C	30 s	
72°C	10 min	
4°C	Hold	

After the PCR was complete, reaction cleanup was performed with AMPure XP beads (Beckman Coulter A63880) and two 80% EtOH wash steps (see Supporting Information for detailed protocol), then the PCR product was eluted into 30 μL low‐TE buffer (10 mM Tris–HCl [pH 8.0] + 0.1 mM EDTA) (note: subsequent experiments that transitioned directly into the dilution step below without a cleanup have also provided good results). An aliquot of the PCR product was then diluted further 1:100 in low‐TE buffer. This dilution ratio can be varied to achieve different proportions of target to natural‐RAD loci in the final dataset (i.e., a 1:1000 dilution of PCR product will reduce the number of target reads compared to a 1:100 dilution; see Section 4). The 1:100 ratio chosen here was expected to produce a relatively high proportion of reads from target loci to facilitate thorough evaluation of primer efficacy.

#### 2.3.3 RAD Library Prep

To generate RAD libraries for both the ampliRAD and traditional RAD sample sets, we performed the following protocol, modified from Ali et al. (2016), simultaneously for both sample sets. This protocol is described with additional details and suggestions in the Supporting Information.

##### 2.3.3.1 Initial Digestion

9 μL of genomic DNA (variable concentrations) and 1 μL of diluted PCR product were combined with 2 μL of digestion master mix (see protocol in Supporting Information). This mixture was incubated at 37°C for 30 min followed by heat inactivation at 80°C for 20 min in a thermocycler.

##### 2.3.3.2 Adapter Ligation

Small aliquots of annealed biotinylated BestRAD *Sbf*I/*Pst*I adapters (50 nM; Ali et al. 2016) were thawed at 4°C in an open thermocycler. While the adapters continued to be held at 4°C, 2 μL of the adapters and 2 μL of ligation mastermix (see protocol in Supporting Information) were added to each well of digested DNA and mixed thoroughly. The adapter aliquots were immediately returned to the freezer. Proper handling of annealed adapters is important because adapter degradation due to many freeze–thaw cycles and extended times at room temperature is a leading cause of poor RAD library generation. The library preparation plate was then incubated at room temperature for 10 min, placed in a refrigerator overnight, then moved to room temperature for 20 min the following morning before the reaction was inactivated with the addition of 2 μL of 0.5 M EDTA to each well. Ali et al. (2016) stopped the ligation reaction via heat inactivation, but we have found stoppage through the addition of EDTA to be more efficient for our workflow. Note, the amount of EDTA added here maintains an inhibitory concentration through the addition of other reagents in the following step.

##### 2.3.3.3 Pooling

After inactivation of the ligase, 8 μL from each well of the library preparation plate was combined into a single 1.5 mL Eppendorf tube. The remaining ligation reactions were frozen and stored for future use. To clean up the DNA, an equal volume of Ampure XP beads was added to the tube and two 80% EtOH washes were performed. The pooled and cleaned DNA was eluted from the beads into 82 μL of low‐TE buffer and placed on a magnet to collect the beads. Finally, 80 μL were transferred to a new tube.

##### 2.3.3.4 DNA Shearing

The original RAD library prep protocol (Ali et al. 2016) mechanically sheared DNA using a Bioruptor NGS sonicator (Diagenode). Here, DNA was enzymatically sheared using NEBNext dsDNA Fragmentase (NEB M0348). To shear, 10 μL of 10X NEBNext dsDNA Fragmentase Reaction Buffer and 10 μL of dsDNA Fragmentase were added to the tube containing 80 μL of pooled DNA. The mixture was immediately vortexed for 3 s, quickly spun down, and incubated at 37°C for exactly 20 min in a thermoblock before the fragmentation reaction was stopped by adding 50 μL of 0.5 M EDTA. To check that the fragmentation reaction had produced sufficient fragments in the desired size range, 1 μL of the reaction mixture was run on an agarose gel and compared to a DNA ladder. The size of fragments generated depends on the amount of time the reaction is allowed to proceed, and the manufacturer recommends experimental optimization of exposure time for a given sample set. In our experience, DNA obtained from samples collected and dried in field‐based settings achieves desired fragment sizes on the shorter end of manufacturer recommendations for fragmentation times.

##### 2.3.3.5 RAD Tag Physical Isolation

Next, the halted fragmentation reaction proceeded directly into the RAD tag physical isolation step without cleanup. Physical isolation of RAD tags from the rest of the genomic DNA was performed as described in Ali et al. (2016). In brief, 20 μL Dynabead M‐280 streptavidin magnetic beads (Life Technologies, 11205D) were washed twice with 100 μL 2X Binding and Wash (B + W) buffer (10 mM Tris–HCl [pH 8.0], 1 mM EDTA [pH 8.0], 2 M NaCl), then resuspended in a volume of 2X B + W buffer equivalent to the final volume of sheared DNA from above (in this case, ~150 μL). The resuspended streptavidin beads were added to the tube of fragmented DNA in order to bind the biotin incorporated onto RAD tags via the biotinylated adapters. The mixture was incubated at room temperature for 20 min with vigorous mixing every 2 min. Next, the tube was placed on a magnet to immobilize the beads and the supernatant was removed. Five wash steps were performed to remove DNA not bound to the streptavidin beads (i.e., non‐RAD tags). For each wash, the beads were resuspended by pipetting in 150 μL 1X B + W buffer, then the tube was placed back on the magnet to immobilize the beads and the supernatant was removed. This was performed three times with room temperature 1X B + W buffer, then twice with 1X B + W buffer heated to 56°C.

To liberate the RAD tags from the streptavidin beads, the beads were washed twice with 100 μL 1X rSmartCut buffer (NEB B6004), then resuspended in 40 μL 1X rSmartCut buffer. 2 μL of SbfI‐HF (NEB R3642) was added, mixed thoroughly, and incubated at 37°C for 60 min with occasional gentle mixing. The streptavidin beads were collected on a magnet and the supernatant containing the liberated RAD tags was moved to a new tube. The liberated DNA was cleaned by addition of an equal volume of Ampure XP beads and two 80% EtOH washes, then eluted in 53 μL of low‐TE buffer. The beads were collected on a magnet and 52 μL of DNA were transferred to a new tube.

##### 2.3.3.6 Final Library Preparation

Sequencing libraries were prepared from the isolated RAD tags with the NEBNext Ultra II DNA Library Prep Kit for Illumina (NEB E7645) according to the manufacturer's protocol. Plate barcodes were supplied with NEBNext Multiplex Oligos for Illumina (96 Unique Dual Index Primer Pairs) (NEB E6440). Adapters were diluted 1:10 as per manufacturer recommendations for low‐input libraries, and size selection was conducted to target insert sizes near 250–300 bp. PCR amplification was performed using 12 cycles. The final library was sequenced on approximately 10% of one NovaSeqX 25B lane using 150 bp paired‐end reads.

### 2.4 Bioinformatics and Analysis

#### 2.4.1 Alignment and Filtering

Custom scripts were used to split raw sequencing reads into fastq files for individual samples based on well barcodes by requiring a perfect match to the barcodes. Residual adapter sequences were trimmed with Cutadapt (Martin 2011), and the reads were aligned to the Chinook salmon reference genome (Otsh_v2.0) (Christensen et al. 2018) with bwa mem (Li 2013) using default parameters.

At this point, PCR duplicates had not yet been removed from the bam files. The initial amplification of target loci in ampliRAD results in PCR products for a given target locus sharing identical start and end positions, and consequently nearly all ampliRAD target data would be eliminated with duplicate removal. Therefore, we created two sets of sorted and indexed BAM files using Samtools (Danecek et al. 2021; Li et al. 2009). One set, retaining all reads, was used for target loci analyses. The second set, processed with Samtools duplicate removal (markdup), was generated for all other analyses. A set of bam files without duplicates removed was also created for the traditional RAD sample set for downstream comparison.

#### 2.4.2 Evaluation of Sequencing and Targeting Success

To evaluate overall sequencing success and the effectiveness of target enrichment, we calculated the following for all samples:

1. Total number of mapped reads (samtools view ‐c ‐F 0x4 sample.bam; Danecek et al. 2021).
2. Total number of reads mapped to the region containing target loci (samtools view ‐c ‐F 0x4 sample.bam NC_056456.1:13402786‐13547912).
3. Percentage of all mapped reads aligned to the target region.

These calculations were performed on the bams prior to duplicate removal (see above). The calculations were repeated for the traditional RAD sampleset for comparison.

#### 2.4.3 Calling Genotypes at Target Loci

To evaluate the efficacy of AmpliRAD for generating genotypes at target loci, we used ANGSD (Korneliussen et al. 2014) to call genotypes at the target SNPs (‐doGeno 4; ‐rf). Genotype likelihoods were calculated under the Samtools model (‐GL 1; Li 2011a), and were used to infer the major and minor alleles (‐doMajorMinor 1) and their frequencies (‐doMaf 3, Kim et al. 2011). A uniform prior was used to estimate posterior genotype probabilities (‐doPost 2). Filtering criteria included a minimum mapping quality score of 30 (‐minMapQ 30), a minimum base quality score of 30 (‐minQ 30), and a posterior genotype probability cutoff of 0.95. These filters were applied to bam files generated prior to duplicate removal. Due to the high read depth at target loci (see Section 3), ANGSD genotype calling proved memory‐intensive. To mitigate this, we implemented a batch processing approach, calling genotypes for all samples in batches of six to eight loci and subsequently concatenating the results. This batch approach was taken in order to thoroughly evaluate all target loci data for the purposes of method validation, but subsampling the data would be a simpler approach for many analyses. The percentage of samples with called genotypes was calculated for each locus. For comparison, we also performed genotype calling on the traditional BestRAD samples using the same ANGSD parameters and batch processing approach. Results were visualized in R (R Core Team 2024) with ggplot2 (Wickham 2016).

#### 2.4.4 Evaluating Evenness of Coverage Across Target Loci

To evaluate read distribution across target loci and assess the evenness of sequencing coverage between target SNPs, we used samtools depth (Danecek et al. 2021) to calculate read depth at each amplicon position. These calculations incorporated base and mapping quality filters of 30 (‐q 30; ‐Q 30). Reads containing deletions were included in the counts (‐J), and only the first read of overlapping read pairs was counted (‐s). For each sample, we calculated the total number of reads covering all target SNPs and subsequently determined the proportion of reads derived from each individual SNP. These proportions were visualized in R (R Core Team 2024; Wickham 2016).

The initial amplification of target loci produces numerous PCR products of identical length for each locus. However, fragmentation during library preparation can influence coverage depth, potentially reducing it near the 3′ amplicon end. This has implications for optimal target SNP placement within amplicons. To assess whether sequencing depth varied along amplicon lengths, we used the depth data generated above. For each target amplicon, we calculated the ratio of read depth at each position to the read depth at the first position. These ratios were then visualized in R (R Core Team 2024; Wickham 2016).

#### 2.4.5 Identifying a Panel of Genome‐Wide RAD SNPs

Beyond targeted sequencing, the AmpliRAD method aims to generate genome‐wide RAD data suitable for population genetic analyses. To assess its efficacy in this context, we identified polymorphic sites across the genome using ANGSD (Korneliussen et al. 2014). This analysis included all 96 AmpliRAD samples but excluded the traditional RAD dataset. The analysis was performed on bam files after removal of PCR duplicates (see above). Filtering criteria were as follows: minimum base and mapping quality scores of 30 (‐minQ 30, ‐minMapQ 30); sites present in at least 50% of individuals (‐minInd 48); a SNP *p*‐value threshold of 1 × 10^−6^ (‐SNP_pval 0.000001; Kim et al. 2011); base quality scores were recalibrated around indels using the SAMtools model (‐baq 1; Li 2011b); per‐site allele frequencies were calculated (‐doMaf 3; Kim et al. 2011) using a uniform prior (‐doPost 2); major and minor alleles were inferred from genotype likelihoods (‐doMajorMinor 1) under the SAMtools model (‐GL 1) (Li 2011a); and a minimum minor allele frequency of 0.05 (‐minMaf 0.05) was applied.

Sequencing depth at each identified SNP was evaluated with samtools depth (Danecek et al. 2021). These calculations incorporated base and mapping quality filters of 30 (‐q 30; ‐Q 30). Reads containing deletions were included in the counts (‐J), and only the first read of overlapping read pairs was counted (‐s).

#### 2.4.6 Genetic Analysis of Dean River Chinook Salmon

To demonstrate the utility of combining targeted and reduced representation sequencing in a single protocol, we employed our ampliRAD dataset to analyze the association of the target loci with a critical adaptive trait and explore population structure in Dean River Chinook salmon.

The target loci in our AmpliRAD dataset reside within and upstream of a gene called *GREB1L* on chromosome 28, a region found to be strongly associated with adult migration timing across numerous populations in the southern portion of the species' range (Hugentobler et al. 2024; Meek et al. 2020; Narum et al. 2018; Prince et al. 2017; N. F. Thompson et al. 2020; T. Q. Thompson et al. 2019; Waples et al. 2022; Willis et al. 2021). However, the association of *GREB1L* with migration timing has not yet been investigated in populations north of the contiguous United States, where migration tends to be more constrained and peak in the summer, as opposed to southern populations where migrations tend to be more extended with peaks in the spring and fall (Waples et al. 2004).

To investigate the association between migration timing and *GREB1L* variation in Dean River Chinook salmon, we analyzed the target SNP genotypes generated above. Because the discovery process and validation of some of the markers have not yet been published, we limited our analyses to three target SNPs previously shown to be associated with migration timing in multiple populations (Supporting Information; Koch and Narum 2020; N. F. Thompson et al. 2020; T. Q. Thompson et al. 2019). Samples were assigned a consensus *GREB1L* genotype (homozygous early, heterozygous, or homozygous late) only if consistent genotype calls were obtained for all three SNPs. These consensus genotypes were used for downstream analyses for both adult and juvenile samples.

Adult samples had been caught near the Dean River mouth, allowing their collection dates to serve as reasonable proxies for the start of their upriver migration. Adult sample collection spanned 70 days, from June 4 to August 12 (Supporting Information). Samples from both collection years (2022 and 2023) were well represented across this span and were analyzed together. To test for an association between *GREB1L* variation and adult migration timing, we divided the sampling period into two 35‐day windows (early season and late season) and tallied the number of early‐ and late‐migrating alleles observed in each window based on the consensus *GREB1L* genotypes. A two‐tailed Fisher's exact test was used to determine if *GREB1L* allele frequencies differed significantly between the early and late season groups. The temporal distributions of each genotype among the adult samples were visualized in R (R Core Team 2024; Wickham 2016).

To explore the relationship between spatial habitat usage and variation at *GREB1L*, we also calculated and compared *GREB1L* allele frequencies in groups of juveniles sampled from different locations in the Dean River basin. The same methods were employed for calling genotypes, evaluating allele frequencies, and testing for significance as described above for the adult samples.

To evaluate population structure in Dean River Chinook salmon, we analyzed the ampliRAD dataset with the genome‐wide SNP panel identified above. Individuals underwent an initial screen for missing data and were excluded if they were missing data at more than 50% of the sites in the panel. Next ANGSD (Korneliussen et al. 2014) was used to perform principal component analysis (PCA). A single read sampling approach was employed to mediate differences in coverage between samples (‐doIBS 1; doCounts 1); quality scores were recalculated around indels (‐baq 1; ‐ref path/to/Otsh_v2.0_genome; Li 2011b); sites with base and mapping qualities below 30 were excluded (‐minQ 30; ‐minMapQ 30); the major and minor alleles were inferred with genotype likelihoods under the samtools model (‐doMajorMinor 1; ‐GL 1; Li 2011a); a covariance matrix was generated with ‐doCov1; and a file was provided to restrict the analysis to loci in the SNP panel (‐rf SNPs.txt). SNPs within the region containing target loci (NC_056456.1:13402786‐13547912) were excluded. The results were visualized in R (R Core Team 2024; Wickham 2016).

### 2.5 Manuscript Generation

This manuscript was conceived, drafted, and finalized by the coauthors of this study. The artificial intelligence tool Google Gemini (version 2.0 Flash) was employed to copy edit and suggest improvements to phrasing in the text.

## 3 Results

### 3.1 AmpliRAD Successfully Generates Data for Target Loci

To evaluate sequencing yields and target region enrichment, we compared read counts between the ampliRAD dataset and the 13 traditional RAD samples (Table 1). The ampliRAD data exhibited a dramatically higher proportion of reads mapping to the target region compared to the traditional RAD data. Specifically, 54.5% of reads mapped to the target region in the ampliRAD dataset, while < 0.01% mapped to the same region in the traditional RAD dataset (Table 1). We conclude ampliRAD successfully enriches for target loci.

**TABLE 1:** Counts of mapped reads.

Data set	Mean number of mapped reads per sample	Mean number of reads mapped to target region per sample	Percent of total mapped reads that mapped to the target region (range across individual samples)
AmpliRAD	5,969,769	3,256,415	54.6% (11.2%–67.5%)
Traditional BestRAD	4,121,662	242	< 0.01% (< 0.01%–0.11%)

**FIGURE 2:**
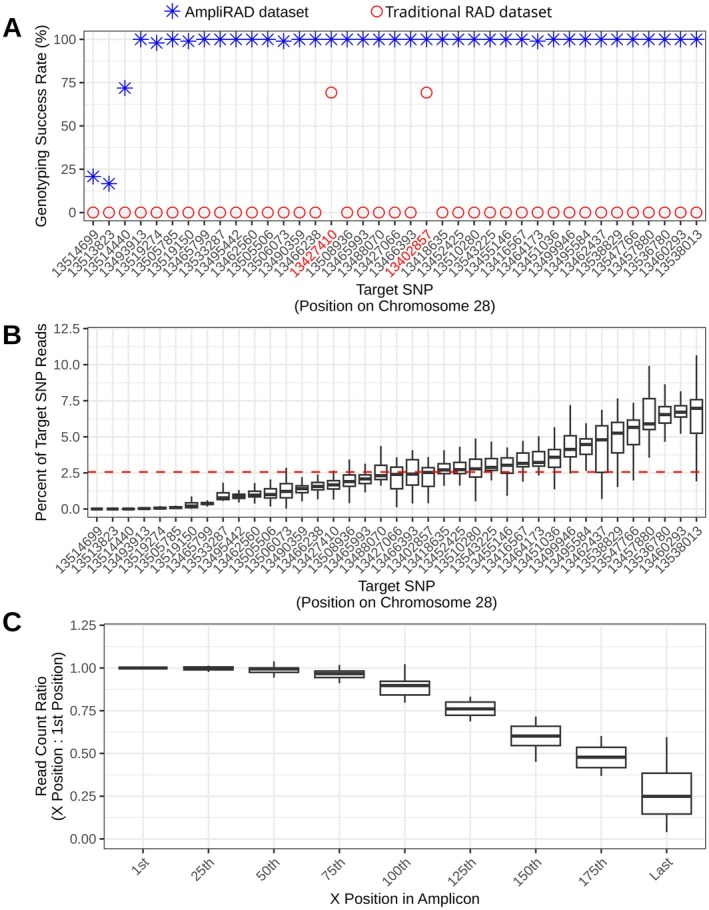
Evaluation of ampliRAD sequencing results. (A) Genotyping success rates for each target SNP in the AmpliRAD and traditional RAD datasets. The two SNPs whose positions are written in red are known to be adjacent to naturally occurring restriction sites. SNP order corresponds to order in part B of the figure. (B) Evenness of coverage across target SNPs in the ampliRAD dataset. The y‐axis indicates the percentage of target SNP reads originating from each target locus. Boxplots indicate the range across individual samples. The red dashed line indicates the expected percentage of reads originating from each locus if coverage was evenly distributed across the 39 targets (1/39). (C) Changes in coverage across the lengths of target amplicons. Boxplots report the ratios between the number of reads at the Xth position in an amplicon and the number of reads at the first position in the amplicon. The “Last” position boxplot corresponds to the ratio between the last position and first position in each target amplicon (target amplicon lengths range from 180 to 325 bp).

The analysis of genotyping success rates for target loci further highlighted the efficacy of ampliRAD as a targeted approach. Thirty‐six of the 39 target SNPs genotyped successfully in over 95% of ampliRAD samples, and 32 SNPs genotyped successfully in 100% of samples (Figure 2A). This high success rate is especially notable given the target primers and PCR conditions had not undergone iteration or optimization after their initial design. Within the traditional RAD dataset, only the two SNPs located adjacent to naturally occurring SbfI restriction sites were successfully genotyped in any of the traditional RAD samples. We conclude that ampliRAD facilitates successful genotyping of target loci that are not represented in traditional RAD datasets.

The analysis examining evenness of coverage across target loci revealed, as expected, a degree of variation in the distribution of target reads across loci. However, the range of variation was narrow for the large majority of loci and samples. Specifically, only a small fraction of loci had less than half or more than three times their expected proportion of target reads (Figure 2B). The targets with the lowest proportions were also those with relatively low genotyping success rates (Figure 2A,B). We conclude the ampliRAD target data demonstrate a relatively even distribution across loci as opposed to being dominated by a small subset of loci.

Analysis of read depth variation along amplicon lengths revealed a decrease in coverage from the beginning to the end of the amplicons (Figure 2C). On average, read depth at the end of amplicons was approximately one‐quarter of that observed at the first position. This decline in coverage is likely influenced by several factors (some of which may be addressed through optimization), including DNA fragmentation time during library preparation, size selection criteria, and the propensity of shorter reads to bind more efficiently to sequencing flow cells. Regardless, these results suggest that, when feasible, target SNPs should be positioned closer to the beginning of the amplicon (i.e., near the primer containing the restriction cut site) than to the end in order to achieve optimal coverage.

### 3.2 AmpliRAD Successfully Generates Data for RAD Loci

Our analyses to identify a panel of genome‐wide RAD SNPs (excluding the target region) resulted in 31,944 loci passing filtering. The number of SNPs identified with RAD data is heavily dependent on factors such as choice of restriction enzyme, species, and filtering criteria, and the number found here is well within a typical range for RAD datasets of this size and scope in Pacific salmon (Prince et al. 2017; T. Q. Thompson et al. 2024). Average coverage per sample at each SNP was 5.6X, which was sufficient for downstream analyses. We conclude ampliRAD successfully generates data for traditional RAD loci (i.e., adjacent to natural restriction sites).

### 3.3 *GREB1L* Variation Is Associated With Temporal and Spatial Distributions in Dean River Chinook Salmon

**FIGURE 3:**
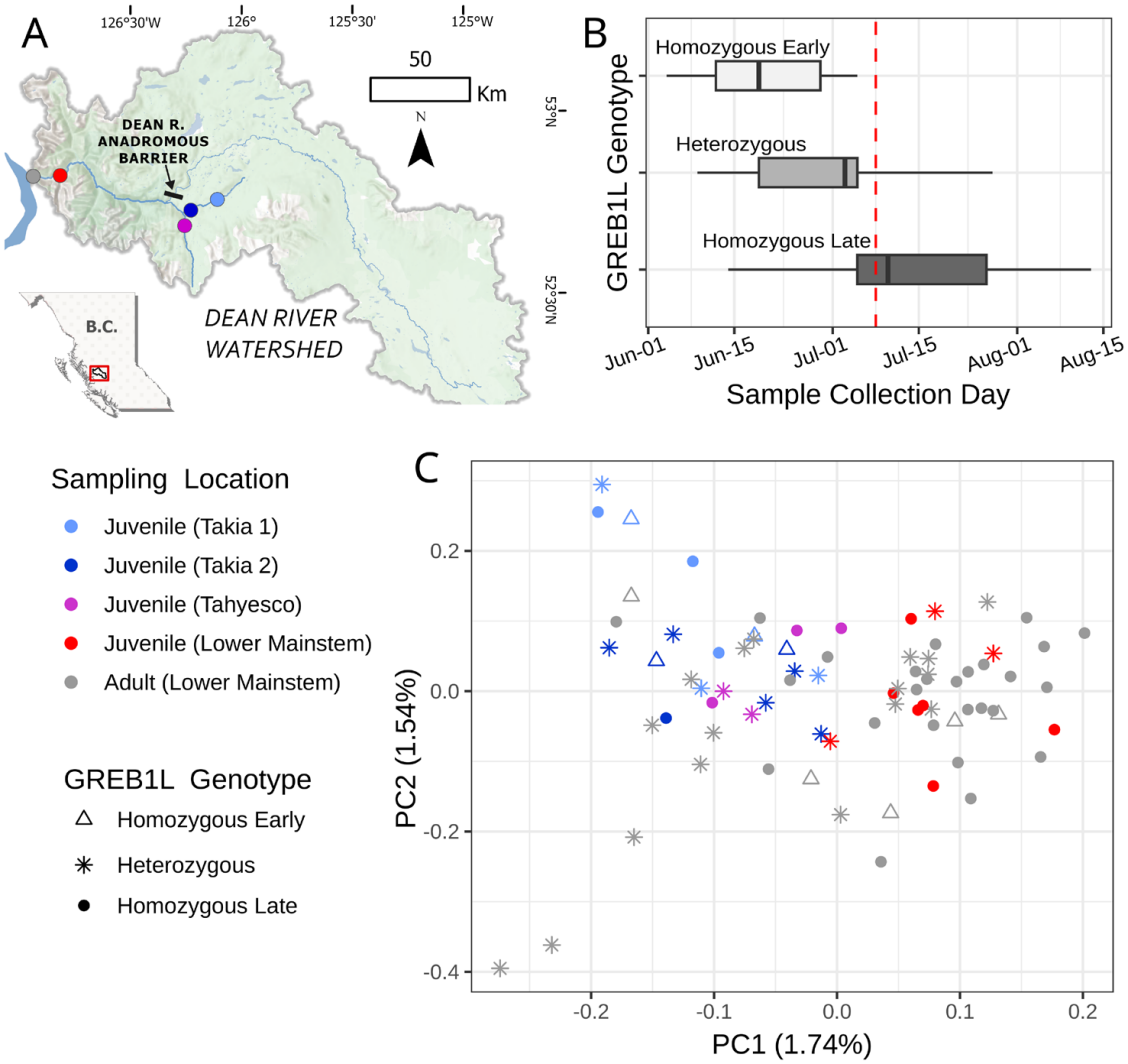
Evaluation of Dean River Chinook salmon. (A) Map of the Dean River Watershed. The circles indicate sample collection locations. The black bar indicates a natural impassable barrier to Chinook salmon located on the Dean River mainstem just upstream of the Takia River confluence. (B) Runtiming of Dean River Chinook salmon by *GREB1L* genotype. The samples used in this analysis were collected at the location indicated by the gray circle in part A. (C) PCA of Dean River Chinook salmon. Color indicates sampling location as indicated in part A, and shape indicates GREB1L genotype call.

Variation at *GREB1L* was clearly associated with adult migration timing in Dean River Chinook salmon. As has been found in southern Chinook salmon populations (Waples et al. 2022), individuals homozygous for the “early” alleles migrated earliest, while those homozygous for the “late” alleles migrated latest, and heterozygotes exhibited an intermediate migration timing (Figure 3B). Heterozygotes showed a greater overlap in migration timing with homozygous “early” individuals than with homozygous “late” individuals, a pattern also reported elsewhere (N. F. Thompson et al. 2020; Waples et al. 2022). The association of GREB1L with migration timing was further supported by a significant difference in *GREB1L* allele frequencies when samples were divided into early and late season groups (Fisher's exact test, *p* < 0.002; Table 2; Supporting Information).

**TABLE 2:** GREB1L allele counts and frequencies among adults caught in the lower Dean River.

	Early season	Late season
Early allele	*n* = 23; freq = 0.37	*n* = 4; freq = 0.10
Late allele	*n* = 39; freq = 0.63	*n* = 38; freq = 0.90

We also observed significant differences in *GREB1L* allele frequencies among juvenile Chinook salmon sampled from different locations within the Dean River watershed (Figure 3A).

Early‐migration allele frequencies were notably lower in the lower mainstem Dean River (0.2) and the Tahyesco (0.15) compared to two separate sampling locations in the upper Takia River (0.6 and 0.55, respectively; Table 3; Figure 3C; Supporting Information). These differences in allele frequencies between each Takia River site and Tahyesco and lower mainstem sites were statistically significant (two‐tailed Fisher's exact test, *p*‐values: 0.01–0.05). Further analysis revealed that these differences were primarily driven by variations in the frequencies of alternative homozygotes (Supporting Information). Although this juvenile dataset does not represent a comprehensive survey of the entire watershed, these findings suggest a relationship between *GREB1L* variation and spatial structure in freshwater habitat use in Dean River Chinook salmon, consistent with observations in more southern watersheds (Waples et al. 2022).

**TABLE 3:** GREB1L allele counts and frequencies among juveniles sampled at four different locations.

	Lower dean	Tahyesco	Takia 2	Takia 1
Early allele	*n* = 4; freq = 0.2	*n* = 3; freq = 0.15	*n* = 12; freq = 0.6	*n* = 11; freq = 0.55
Late allele	*n* = 16; freq = 0.8	*n* = 17; freq = 0.85	*n* = 8; freq = 0.4	*n* = 9; freq = 0.45

### 3.4 Principle Component Analysis of Dean River Chinook Salmon

Of the 96 ampliRAD samples, 78 (81%) passed quality filtering for inclusion in the principal component analysis (Supporting Information). The samples that failed filtering were excluded due to excessive missing data at loci within the SNP panel (see Section 2; Supporting Information). Given that the quantities of genomic DNA were not normalized across samples prior to library preparation, this sample drop out rate is not excessive. It could likely be reduced further by normalizing the initial genomic DNA inputs as well as by decreasing the ratio of target amplicons in the input DNA (see Table 1).

Despite an overall weak genetic structure, we observed clear patterns related to geographic location in our dataset (Figure 3C). Most adults clustered with juveniles sampled from the lower Dean River, while juveniles from the Takia and Tahyesco Rivers clustered together, along with a small subset of adults. While the limited spatial sampling of juveniles likely precludes a comprehensive assessment of population structure in Dean River Chinook salmon, these results reveal spatial genetic relationships that warrant future investigation. Moreover, by overlaying *GREB1L* genotype data onto the PCA, Figure 3C demonstrates the utility of ampliRAD to integrate targeted genotype data with genome‐wide data in a single analysis, revealing the distribution of target genotypes within the broader genetic context.

## 4 Discussion

AmpliRAD represents, to our knowledge, the first method combining both targeted and broad reduced‐representation sequencing, effectively merging the benefits of both approaches through the addition of a single multiplex PCR step. This streamlined process allows for simultaneous generation of genome‐wide RAD data and targeted genotyping at specific loci of interest, even those not adjacent to natural restriction sites. In addition to presenting ampliRAD and evaluating its efficacy, we demonstrate its utility to address diverse biological questions by analyzing Dean River Chinook salmon migration timing and spatial habitat use. With ampliRAD data, we evaluated population structure using tens of thousands of RAD SNPs while concurrently analyzing genotypes at adaptive markers not located near restriction sites.

Beyond advances in sequencing technology, innovations in library preparation methods are democratizing molecular biology, making genomic analysis accessible to researchers with limited resources or equipment. The core innovation of AmpliRAD, the integration of targeted and genome‐wide approaches in a single workflow, continues this trend. By enabling researchers to assay both specific candidate loci of interest and genome‐wide patterns of variation simultaneously, ampliRAD eliminates the need to generate multiple types of data using different protocols when both targeted and genome‐wide information are required. Additionally, the modified RAD protocol we employed, building upon Ali et al. (2016), further enhances accessibility for reduced‐representation library preparation. While maintaining the original protocol's simplicity (e.g., early plate consolidation) and efficiency, we replace the bioRuptor‐based sonication with enzymatic shearing, streamlining the process and eliminating the need for sonication equipment.

Repetitive elements or low complexity regions of genomes present substantial challenges for targeted sequencing applications (e.g., due to the difficulty of specific primer design), yet may contain important loci of interest (Liao et al. 2023). AmpliRAD addresses this issue by leveraging paired‐end sequencing which, when combined with alignment to a high‐quality reference genome, can facilitate accurate genotyping of otherwise challenging loci so long as target and off‐target reads align best to their native regions. This proved useful for our analysis of variation near *GREB1L*. With ampliRAD, we successfully genotyped target loci within a section of this region where the difficulty of designing specific primers has hindered targeting with other methods (Supporting Information). However, this section also contained the three loci with low genotyping success rates, and aligned read counts tended to be lower than for target loci from other sections (Figure 2B, Supporting Information). It therefore remains advisable to design highly specific primers when possible, and anticipate a higher failure rate and need for deeper sequencing when targeting difficult regions. Careful validation of genotyping performance for a given target panel also remains important, especially when primer specificity is suboptimal.

The primer pairs used here were implemented without iterative redesign, and their success and relative evenness of coverage across loci support the robustness of the ampliRAD protocol. This is especially noteworthy given that multiplex PCR applications often require extensive primer optimization, which is time‐intensive. However, our results also indicate that designing target amplicons with longer lengths may be beneficial. The decrease in coverage from the beginning to the end of the amplicons was steeper than we anticipated (Figure 2C), and shorter read lengths may have impaired mapping efficiency and reduced read counts for targets in repetitive regions. Therefore, there may be a benefit to employing larger amplicon size parameters during primer design (perhaps 300–450 bp, positioning the target SNP on the first read), along with complementary size selection criteria during library preparation. This may enhance performance, particularly in challenging genomic regions. Researchers aiming for larger sequencing insert sizes should also ensure their amplicon lengths are complementary to the intended insert sizes in order to mitigate the preferential sequencing of shorter fragments.

A key component that can be optimized based on the circumstances and goals of specific labs is the dilution ratio of the multiplex PCR product (see Section 2), which influences the proportion of target to natural‐RAD loci in the final dataset (i.e., a 1:1000 dilution of PCR product will reduce the number of target reads compared to a 1:100 dilution). An optimal dilution ratio will depend on factors such as the goals of the researcher, the genome size of the assayed organism, the expected number of natural restriction sites in the genome, the concentration and quality of the genomic DNA, and the depth of sequencing per sample that will be performed. In this study, we employed a 1:100 dilution ratio to deliberately ensure an excess of amplicon DNA, allowing for an unambiguous evaluation of primer efficacy. We then sequenced relatively deeply to maintain adequate coverage at natural RAD loci for population structure analyses. While the resulting data robustly addressed our research goals, reducing the ratio of target reads in the data would substantially increase sequencing efficiency (see Table 1). We have subsequently implemented a 1:1000 dilution in other experiments with good results. While the dilution ratio can be optimized to increase efficiency for the circumstances of specific labs or projects, a 1:1000 or 1:100 ratio coupled with relatively deep sequencing will, in many situations, represent a reliable starting point for initial assessments.

Finally, we leveraged ampliRAD data to address a timely biological question: the association of *GREB1L* variation with adult migration timing and spatial distribution in Dean River Chinook salmon. While this association has been well‐documented in southern Chinook populations (Waples et al. 2022), its relevance in northern populations, which exhibit different migration timing patterns, had not yet been explored. Understanding *GREB1L* variation and its influence on migration timing in northern rivers is crucial, especially in the context of a warming climate that may cause seasonal streamflow and temperature patterns in northern rivers to more closely resemble those in the south (Crozier et al. 2021; Crozier and Siegel 2025). Our results clearly demonstrate an association of *GREB1L* with migration timing in Dean River Chinook and reveal significant allele frequency differences among juveniles from different parts of the watershed, suggesting spatial habitat use may be structured by adult migration timing. These findings highlight the potential for ampliRAD to contribute valuable insights to conservation and management efforts by simultaneously facilitating analyses of adaptive variation and population structure.

## 5 Conclusion

For studies requiring both targeted genotyping and genome‐wide SNP data, ampliRAD offers a novel solution. By combining the strengths of targeted and reduced‐representation sequencing, this method enables efficient analysis of both specific loci and broad genomic patterns. Our updated RAD protocol, incorporating enzymatic shearing, simplifies library preparation and enhances accessibility. AmpliRAD is particularly well‐suited for projects where whole‐genome sequencing is unnecessary, but purely targeted approaches are insufficient.

## Author Contributions

**Tasha Q. Thompson:** conceptualization (lead), data curation (lead), formal analysis (lead), investigation (lead), methodology (lead), project administration (supporting), visualization (lead), writing – original draft (lead), writing – review and editing (lead). **Michael R. Miller:** conceptualization (supporting), methodology (supporting), writing – review and editing (supporting). **Matthew R. Sloat:** conceptualization (supporting), funding acquisition (lead), investigation (supporting), project administration (lead), supervision (lead), visualization (supporting), writing – review and editing (supporting).

## Funding

This work was supported by Paul G. Allen Family Foundation, G‐202312‐14332 and the Jack Polsky Conservation Research Fellowship.

## Disclosure

Benefit Sharing: This research was conducted in partnership with Heiltsuk Nation and Nuxalk Nation under research protocol agreements. The genetic insights generated from this study, particularly regarding *GREB1L* variation and migration timing in Dean River Chinook salmon, will be shared with Indigenous communities and relevant fisheries management agencies to support conservation and stewardship efforts. The AmpliRAD methodology developed here is made freely available to the scientific community, and the protocol is provided in supplemental materials to facilitate adoption by researchers working on the conservation of salmon or other species. All data used in this study are also available for public use.

## Conflicts of Interest

The authors declare no conflicts of interest.

## Supporting information


**Data S1:** ece372990‐sup‐0001‐Supinfo.pdf.

**Data S2:** ece372990‐sup‐0002‐Supinfo.xlsx.

## Data Availability

All raw sequencing data is available on NCBI with BioProject accession number PRJNA1262529. All scripts and other data files used for analysis have been uploaded to the Dryad Data Repository (Dataset: https://doi.org/10.5061/dryad.d51c5b0fb).
