# Supplementary material for: AmpliRAD: A New Method Combining Amplicon and RAD Sequencing
Source: Ecol Evol. 2026 Feb 11;16(2):e72990. doi: 10.1002/ece3.72990 (PMC12893785; doi:10.1002/ece3.72990)
Supplement: Supplementary file 1 — Data S1: ece372990‐sup‐0001‐Supinfo.pdf. [file ECE3-16-e72990-s002.pdf]

# AmpliRAD Protocol

---

## 1 – Amplification of Target Loci

**Materials:**
–Genomic DNA
–Target loci primers with attached restriction sites
–Low TE buffer (10 mM Tris-HCl [pH 8.0], 0.1 mM EDTA)
–Platinum$^{TM}$ Multiplex PCR Master Mix (Applied Biosystems 4464268)
–80% EtOH (freshly prepared)

### 1.1 – Make a working stock of pooled primers (final: 0.5 µM each)

*This assumes that primers have not yet been pooled and are at their original concentration of 100 µM.*

*The following makes 4600 µL– enough for 20 plates (with extra to account for pipetting error).*

1) Combine the following in a 5 mL (or larger) tube and mix:

    23 µL   Each Primer (100 µM starting concentrations)
     X µL   Low TE buffer (X = 4600 - (23 * number of primers))
    =================================================
    Optional: aliquot mixture into 20 tubes of 230 µl each

## 1.2 – Multiplex PCR to amplify target loci

2) Prepare the multiplex PCR reactions:

   Per rxn:
   Combine the following in individual wells and mix.

   5 μL Platinum™ Multiplex PCR Master Mix
   2 μL Primer working stock (see above)
   3 μL Genomic DNA
   ======================
   10 μL total

*******OR*******

   Per plate (96 rxns + extra):
   Combine the following in a 1.5 mL tube and mix.

   550 μL Platinum™ Multiplex PCR Master
   220 μL Primer working stock (see above)
   =================================

   Add 7 μL of the above mixture to each well of a 96-well plate.
   Next, add 3 μL Genomic DNA to each well and mix.

3) Seal the reaction plate, place in a thermocycler with heated lid on, and run the following program:

| | | |
|---|---|---|
| 95°C | 5 min | |
| ------------------------------- | | |
| 95°C | 30 sec | |
| 57°C | 90 sec | (35 cycles) |
| 72°C | 30 sec | |
| ------------------------------- | | |
| 72°C | 10 min | |
| 4°C | Hold | |

4) Clean up the multiplex PCR reaction:
    a) Add 10 µL of well-mixed room temp Ampure XP beads to each well….Mix thoroughly….Incubate 5 min at room temp….Place on magnet 5 min or until solution is clear….Carefully remove and **discard supernatant** (don't disturb beads)

    b) While the plate is still on the magnet, wash the beads twice with 150 µL freshly prepared 80% EtOH. During each wash, incubate beads in EtOH for 30 seconds (but not longer).

    c) After washes are complete, allow beads to air dry at room temperature (typical time is <5 min: beads should still be shiny and not exhibit any cracking).

    d) Remove plate from magnet and resuspend beads in 20-100 µL low TE buffer….Mix thoroughly….Incubate 5 min at room temp….Place plate on magnet for 5 min or until solution is clear….Carefully **transfer supernatant** to a new plate.

5) Run PCR product on gel to verify DNA present in anticipated amplicon size range.

6) Dilute the PCR product in Low TE buffer to prepare for RAD library preparation.
    a) Quantification after dilution is recommended.

*****Continue below for RAD library prep*****

# 2 – RAD Preparation

**Materials (ensure these are on hand prior to library prep):**
–Genomic DNA
–Diluted target amplicons (see above)
–Low TE buffer (10 mM Tris-HCl [pH 8.0], 0.1 mM EDTA)
–DNA-grade Water
–SbfI-HF (NEB R3642) or PstI (NEB R0140)
–10X rCutSmart buffer (NEBuffer 4 may be used instead)
–rATP (100 mM; Fermentas R0441)
–T4 DNA Ligase (400,000 units/ml; NEB M0202L)
–Annealed BestRad SbfI/PstI adaptors (50 nM for SbfI digests or 1 uM for PstI digests)
–0.5M EDTA
–Ampure XP beads
–80% EtOH (freshly prepared)
–dsDNA Fragmentase (NEB M0348)
–dsDNA Fragmentase reaction buffer (10x)
–Dynabeads M-280 streptavidin magnetic beads (Invitrogen 11206D)
–2X Binding and Wash (B+W) Buffer (10 mM Tris-HCl [pH 8.0], 1 mM EDTA [pH 8.0], 2 M NaCl)

–NEBNext Ultra II DNA Library Prep Kit for Illumina (NEB E7645)
–NEBNext Unique Dual Index Primers (NEB E6440)

## 2.1 – Digestion and RAD adapter ligation

7) Normalize genomic DNA between samples if desired.

8) Prepare Digestion Master Mix:
For one plate (volumes are adjusted for pipetting error)
Combine the following in a 1.5 mL tube and mix:

| | |
|---|---|
| 85.68 ul | Water |
| 151.2 ul | **10X** rCutSmart buffer |
| 15.12 ul | SbfI-HF **or** PstI |

===========================

9) Into each 96 plate well, pipet 9 uL genomic DNA and 1 uL diluted target amplicons (this ratio can be varied, and may also include the addition of water, but should add up to 10 uL total).

10) Into each well, pipet **2 ul Digestion Mater Mix** and mix thoroughly. Seal plate when finished.

11) Incubate plate at 37°C for 30 minutes (mix/shake occasionally) followed by heat inactivation at 80°C for 20 minutes (thermocycler).

12) Prepare Ligation Master Mix:

    <u>For one plate (volumes are adjusted for pipetting error)</u>

    Combine the following in a 1.5 mL tube and mix:

    | | |
    |---|---|
    | 161.28 ul | Water |
    | 49.92 ul | rCutSmart buffer |
    | 20.16 ul | rATP (100 mM) |
    | 20.16 ul | T4 DNA Ligase |

    ======================

13) Thaw a fresh aliquot of annealed BestRAD SbfI/PstI adapters at 4°C in an open thermocycler or on ice. Mix and spin briefly, then return to the cold thermocycler or ice.
    a) While the adapters are held at a cold temperature, add **2 ul annealed** BestRad SbfI/PstI adaptors (50 nM for SbfI digests or 1 uM for PstI digests) to each well of the digested DNA plate.
    b) Immediately seal the adapter plate and return to freezer if it will be used again.

14) Into each well of the digested DNA plate, pipet **2 ul Ligation Master Mix** and mix thoroughly. Seal the plate.

15) Incubate plate at room temperature for 10 minutes, place in the fridge (~4°C) overnight, then at room temperature for about 20 minutes in the morning.

**End Day 1.**

====================================================================

================================================================================

**Next Day:**

16) After ligation plate has been moved to room temperature for about twenty minutes, inactivate ligation reaction by adding 2 ul 0.5M EDTA to each well (mix by pipetting).

17) Plate Pooling (MAKE SURE YOU'VE INACTIVATED LIGASE FIRST!!!!):
    a) Use a multichannel pipette to transfer 8 ul from each 96 plate well into a sterile trough.
    b) Transfer pooled library from trough to a 1.5 ml tube.
    c) Store plate with remaining digested/ligated DNA for future multiplexing. Label appropriately.

18) Pooled DNA cleanup:
    a) Add an equal volume of well-mixed room temp Ampure XP beads to the tube of pooled DNA….Mix thoroughly….Incubate 5 min at room temp….Place on magnet 5 min or until solution is clear….Carefully remove and **discard supernatant** (don't disturb beads)

    b) While the tube is still on the magnet, wash the beads twice with 800 µL freshly prepared 80% EtOH. During each wash, incubate beads in EtOH for 30 seconds (but not longer).

    c) After washes are complete, allow beads to air dry at room temperature (typical time is <10 min: beads should still be shiny and not exhibit any cracking).

    d) Remove tube from magnet and resuspend beads in **82 uL Low TE buffer**….Mix thoroughly….Incubate 5 min at room temp….Place plate on magnet for 5 min or until solution is clear….Carefully **transfer 80 uL supernatant** to a new 1.5 mL tube.

****Possible stop point***

## 2.2 – Enzymatic Shearing with dsDNA Fragmentase

19) Prepare Fragmentation Reaction
*Rxn begins as soon as Fragmentase is added–**work quickly and be precise with time**.*

a) Preheat thermoblock to 37°C

b) To the tube containing 80 uL ligated DNA, add:
10 uL 10X Fragmentase Reaction Buffer
10 uL dsDNA Fragmentase (vortex enzyme for 3 sec right before)
=====================================================

c) Vortex tube for 3 seconds and quickly spin down

20) Incubate the tube at 37°C in the thermoblock for the chosen time (18-23 minutes).
a) Stop the reaction by adding **50 uL 0.5M EDTA as soon as the time is reached.**

21) Run 1 ul on gel to check shearing efficiency (no cleanup needed if running on agarose gel)--a wide smear will be present, but density should be centered around desired size range (about 300 bp)--check manufacturer protocol for examples.

*****Possible Stop Point*****
Or
*****Proceed directly into next step (no cleanup needed)*****

## 2.3 – RAD Tag Physical Isolation

22) Prepare Binding and Wash (B+W) buffer aliquots (see Materials above)

    a) Prepare 1 mL **1X** B+W buffer by mixing:

| | |
|---|---|
| 500 uL | **2X** B+W buffer |
| 500 uL | Water |

==========================

    b) Divide into two 500 uL aliquots
        i) Keep one at room temperature
        ii) Heat the other to 56°C (e.g., in a thermoblock)

23) Prepare Dynabead M-280 streptavadin magnetic beads

    a) Transfer 20 ul (40 ul for PstI libraries) well-mixed Dynabeads to a 1.5 ml tube.

    b) Place the tube on a magnet until clear and **remove the supernatant**.

    c) Wash the beads with 100 uL **2X** B+W buffer….Mix 30 sec….Quick spin….Magnet 5 min or until clear….Remove supernatant….REPEAT for a total of 2 washes.

    d) Resuspend beads in 150 ul **2X** B+W buffer.

24) Bind RAD tag DNA to Dynabeads
    a) Add the 150 uL of washed Dynabeads to the 150 uL of fragmented DNA from the shearing step (the B+W buffer will then be at 1X).
    b) Incubate at room temperature for 20 min (keep beads suspended–mix every 2 minutes or place on shaker)

25) Wash RAD tags to remove off-target DNA
    a) Vortex briefly….Quick spin….Magnet for 5 min or until clear….Remove supernatant
    b) Resuspend beads with 150 ul **room temp 1X** B+W buffer….Mix by pipetting...Magnet until clear…Remove supernatant
        i) Repeat for a total of **3 room temp** washes
        ii) Repeat washes **2 more times with 56°C 1X** B+W buffer
        iii) When washes are complete, remove supernatant, leave beads on magnet

26) Liberate RAD tags from Dynabeads

a) Prepare **1X** rCutSmart buffer by combining:

| | |
|---|---|
| 30 uL | **10X** rCutSmart buffer |
| 270 uL | Water |

==========================

b) Resuspend the final washed Dynabeads (still bound to RAD tags) with 100 uL **1X** rCutSmart buffer…Mix by pipetting….Magnet until clear….Remove supernatant.

i) Repeat for a total of 2 washes

c) Resuspend the Dynabeads (still bound to RAD tags) with 40 uL **1X** rCutSmart buffer

d) Add 2 uL *SbfI*-HF (use for both *SbfI* and *PstI* libraries) to the resuspended Dynabeads....Mix thoroughly.

e) Incubate tube at 37°C for 60 minutes with occasional gentle mixing.

f) Quick spin….Magnet until clear….**Keep supernatant** by transferring to a new 1.5 mL tube. The supernatant contains the liberated RAD tags.

27) DNA cleanup:

a) Add an equal volume (~43 uL) of well-mixed room temp Ampure XP beads to the tube of pooled DNA….Mix thoroughly….Incubate 5 min at room temp….Place on magnet 5 min or until solution is clear….Carefully remove and **discard supernatant** (don't disturb beads).

b) While the tube is still on the magnet, wash the beads twice with 200 µL freshly prepared 80% EtOH. During each wash, incubate beads in EtOH for 30 seconds (but not longer).

c) After washes are complete, allow beads to air dry at room temperature (typical time is <5 min:  beads should still be shiny and not exhibit any cracking).

d) Remove tube from magnet and resuspend beads in 53 uL Low TE buffer….Mix thoroughly….Incubate 5 min at room temp….Place plate on magnet for 5 min or until solution is clear….Carefully **transfer 52 uL supernatant** to a new 1.5 mL tube.

*****Possible stop point*****

## 2.4 – Sequencing Library Preparation

28) Use NEBNext Ultra II DNA Library Prep Kit for Illumina (NEB E7370S/L) and follow manufacturer's protocol with the following modifications:
    a) Use 1:10 diluted adapter for **SbfI** libraries (no dilution for PstI libraries).
    b) We always perform size selection. Prior to size selection, we measure the library volume. Typically, more water is required to bring the volume up to 100 ul than is suggested in the NEB instructions.
    c) We typically use ~20% of the template to perform a test PCR using 15 cycles (lower for PstI or libraries using high DNA inputs) and run the product on a fragment analyzer to determine yield. A second (final) PCR is then performed with the remaining template using fewer cycles informed by the fragment analyzer results (typically 6-12, depending on DNA quantity, quality, and restriction enzyme choice).
